# ‘The medicine is not for sale’: Practices of traditional healers in snakebite envenoming in Ghana

**DOI:** 10.1371/journal.pntd.0009298

**Published:** 2021-04-16

**Authors:** Jonathan Steinhorst, Leslie Mawuli Aglanu, Sofanne J. Ravensbergen, Chrisantus Danaah Dari, Kabiru Mohammed Abass, Samuel Osei Mireku, Joseph Ken Adu Poku, Yeetey A. K. Enuameh, Jörg Blessmann, Robert A. Harrison, John H. Amuasi, Ymkje Stienstra

**Affiliations:** 1 University of Groningen, University Medical Center Groningen, Department of Internal Medicine/Infectious Diseases, Groningen, The Netherlands; 2 Kumasi Center for Collaborative Research, Global Health and Infectious Diseases Group, Kumasi, Ghana; 3 Ghana Health Service, Upper West Region- Wa, Ghana; 4 Presbyterian Hospital Agogo, Ghana; 5 School of Public Health, Kwame Nkrumah University of Science and Technology, Kumasi, Ghana; 6 Bernhard Nocht Institute for Tropical Medicine, Department of Infectious Disease Epidemiology, Hamburg, Germany; 7 Liverpool School of Tropical Medicine, Centre for Snakebite Research and Interventions, Liverpool, United Kingdom; Universidad de Costa Rica, COSTA RICA

## Abstract

**Background:**

Snakebite envenoming is a medical emergency which is common in many tropical lower- and middle-income countries. Traditional healers are frequently consulted as primary care-givers for snakebite victims in distress. Traditional healers therefore present a valuable source of information about how snakebite is perceived and handled at the community level, an understanding of which is critical to improve and extend snakebite-related healthcare.

**Method:**

The study was approached from the interpretive paradigm with phenomenology as a methodology. Semi-structured interviews were conducted with 19 traditional healers who treat snakebite patients in two rural settings in Ghana. From the Ashanti and Upper West regions respectively, 11 and 8 healers were purposively sampled. Interview data was coded, collated and analysed thematically using ATLAS.ti 8 software. Demographic statistics were analysed using IBM SPSS Statistics version 26.

**Findings:**

Snakebite was reportedly a frequent occurrence, perceived as dangerous and often deadly by healers. Healers felt optimistic in establishing a diagnosis of snakebite using a multitude of methods, ranging from herbal applications to spiritual consultations. They were equally confident about their therapies; encompassing the administration of plant and animal-based concoctions and manipulations of bite wounds. Traditional healers were consulted for both physical and spiritual manifestations of snakebite or after insufficient pain control and lack of antivenom at hospitals; referrals by healers to hospitals were primarily done to receive antivenom and care for wound complications. Most healers welcomed opportunities to engage more productively with hospitals and clinical staff.

**Conclusions:**

The fact that traditional healers did sometimes refer victims to hospitals indicates that improvement of antivenom stocks, pain management and wound care can potentially improve health seeking at hospitals. Our results emphasize the need to explore future avenues for communication and collaboration with traditional healers to improve health seeking behaviour and the delivery of much-needed healthcare to snakebite victims.

## Author summary

Many snakebites in tropical lower and middle-income countries are treated at the community level by traditional healers whose services are often accessible, affordable and offer care within the realms of local tradition and culture. A drawback of traditional care, however, is the associated delay or non-presentation of patients in need of antivenom and medical management at hospitals. We conducted interviews with traditional healers in two separate areas of rural Ghana to chart the socio-cultural context, perceived problems, treatment approaches, health seeking behaviors, and possibilities for future collaboration in snakebite treatment and management between hospitals and traditional healers. Healers described a holistic treatment approach, addressing physiological, psychological, and spiritual complications of snakebite. Healers reported high success rates with therapies ranging from herbal medicine to minor surgical interventions. Major factors precluding hospital health seeking of patients were identified as costs, poor accessibility, lack of antivenom and insufficient pain control. Gaps in clinical management are modifiable factors that, if improved, are likely to increase clinical health seeking. Our findings emphasize that traditional healers can provide a critical link between snakebite patients and hospitals, making their involvement crucial to the rural expansion of snakebite research and emergency care.

## Introduction

Snakebite envenoming, designated a priority neglected tropical disease by the World Health Organisation (WHO), can cause serious and potentially fatal toxicity and disability to the human victims [1]. Depending on the type of snake and the degree of venom exposure, the range of clinical symptoms spans local and systemic cytotoxicity, neurotoxicity, haemotoxicity as well as cardio- and nephrotoxicity [2,3]. Though largely unrecognized, the yearly global incidence of snakebite is estimated at up to 5.4 million bites, of which 2.7 million are venomous, resulting in 81 000–138 000 deaths annually [4]. Based on estimates from hospital admissions [5,6], the incidence of snakebite in Ghana ranges from 50-110/100 000 population and a national average of 310 deaths annually [7], although these data are almost certainly a gross underestimation, since many snakebite victims never present to hospitals [5,7,8]. The disease burden of snakebite in West Africa alone is estimated to amount to ~320 000 disability-adjusted life years, the largest driver of which are years of life lost (~290 000) [7]. The rural poor in Sub-Saharan Africa, South Asia and South-East Asia are disproportionally affected, as the most venomous snakes coexist with them in the areas they live and work in [5,8,9]. Bites are incurred most frequently by young and economically active members of communities aged 15–50 years [5,10] and are most often localized on the extremities [9,10].

The most effective treatment of systemic snakebite envenoming consists of rapid transport to a hospital where prompt administration of antivenom, and where necessary cardiovascular, respiratory supportive therapy and surgical debridement of necrosed tissue or even amputation can be delivered [3,11,12]. In impoverished rural settings, however, long distances to health facilities coupled with scanty knowledge and scepticism of allopathic medicine affects treatment seeking behaviour [13,14]. In addition, treatment at a hospital is costly- wholesale costs for an effective dose of antivenom in Sub- Saharan Africa can range from $ 55–640, though the costs borne by patients can vary considerably depending on national and regional procurement strategies [15]. Transportation, hospitalization, foregone income and expenses for care-takers further aggravate the economic burden [13,16]. The pursuit of biomedical treatment may frequently be in vain, as antivenoms are sometimes not in stock due to their high cost and short shelf lives, or hospitals stock ineffective antivenoms [15,17]. For these reasons, patients may have little hope for positive outcomes in seeking treatment at the hospital and instead turn to traditional healers (TH) [13,18].

Traditional medicine according to the World Health Organisation ‘refers to knowledge, skills and practices based on the theories, beliefs and experiences indigenous to different cultures, used in the maintenance of health and in the prevention, diagnosis, improvement or treatment of physical and mental illness’ [19]. The preferential consultation of THs in many developing countries stems from their distribution and accessibility, their embedment into local culture and society as well as their affordability [18,20]. In Ghana, a TH might serve an average population of 400 individuals [21] whereas the ratio of university-trained physicians to population is only approximately 1.2/10 000 with significant interregional variation [22]. A household survey in northern Ghana showed that THs tend to nearly half of all snakebite victims in the community [10], a finding similar to a survey of snakebite victims in Sri Lanka, which showed that 43% of victims first sought aid from a TH [18].

The snakebite treatment repertoire of THs described so far in literature encompasses a variety of different practices ranging from the administration of topical and emetic herbal concoctions, snake stones, tourniquets, all the way to cutting and suction of the bite wound [3,13,20,23,24]. These techniques and applications have not only been shown to be woefully ineffective, but they can even cause outright harm and worsen health outcomes [3,11,25]. Some interventions of THs only aggravate and prolong the suffering inflicted on patients through venomous bites, increasing the chances of long-term organ damage, extensive tissue necrosis, amputations and the accompanying psychosocial and socio-economic implications [9,26]. Perhaps, the most unique feature of traditional medicine is that it draws upon mystical-spiritual explanations for causes of injuries and disease [27–29]. A common belief, for example, is that disease and snakebites can be related to witchcraft and curses, but also personal and social disobedience or sinful behaviours [27,29,30].

THs can therefore serve as a gateway to charting the ethnic, cultural, social, and epidemiological dimensions of snakebite envenoming. This study seeks to comprehensively describe snakebite envenoming at the community level from the perspective of the traditional healer by means of interviews with THs in two regions in Ghana.

## Methods

### Ethics statement

Ethical clearance was obtained from the Committee on Human Research and Publication Ethics, Kwame Nkrumah University of Science and Technology and from the Medical Ethical Committee of the University Medical Centre Groningen, The Netherlands (CHRPE/AP/354/19). Written informed consent was obtained from THs after being briefed about the goals and the procedure of the study and participation was entirely voluntary. After completion of the interview, THs were offered between 10–30 Ghana Cedis (~ 1,50–5,00 €) as compensation for their time and any transportation costs incurred (the national daily minimum wage in Ghana is 11.82 Ghana Cedis [31]).

### Study design

An interpretative paradigm with a phenomenological approach was employed as the study methodology. This approach elicited the stories of THs expressed through interviews. By studying the social and cultural variables that impact on practices in the management of snakebite and the interplay between these factors [32], we sought to understand the practices of THs through different interpretations arising from their social interactions. Attention was given to the assumptions outlined by Grossoehme [33] for phenomenology research. This design was deemed ideal for defining what the experiences of THs mean in relation to practices of snakebite management and health seeking behaviors of snakebite victims.

### Study location

**Fig 1:**
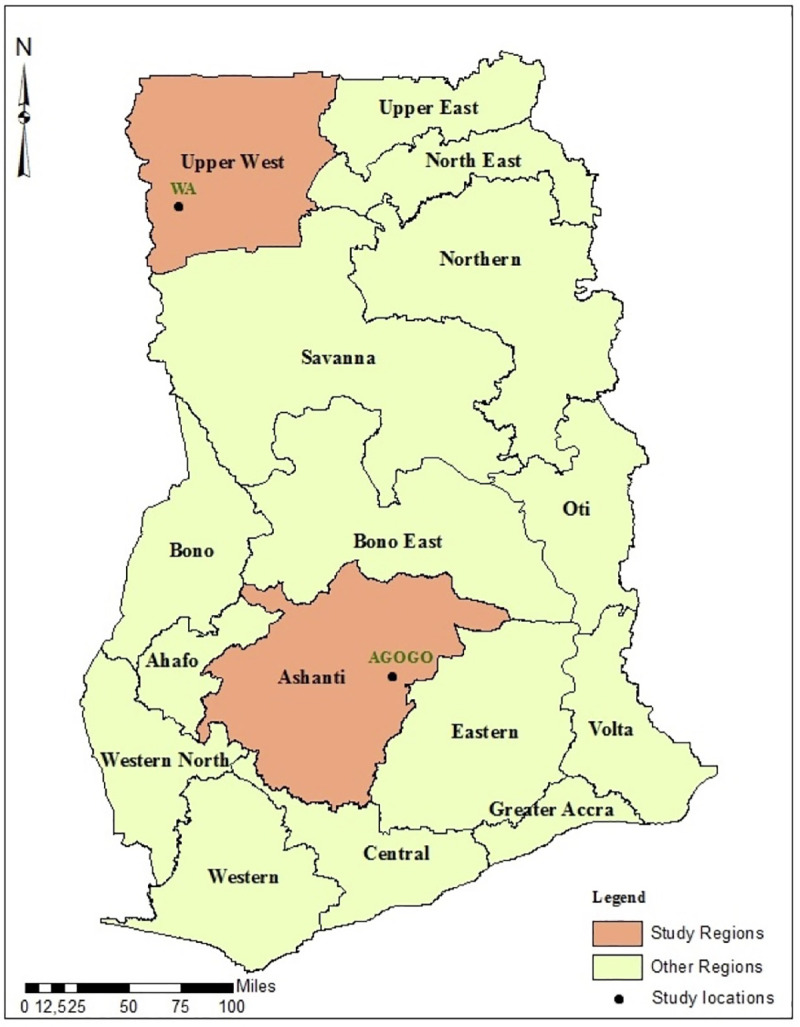
Study regions in Ghana (Created using ArcGIS® software version 10.5.1, ESRI Inc., Redlands, CA, U.S.A).

Interviewees were identified through purposive sampling in areas surrounding the towns of Agogo and Wa in the Ashanti Region and Upper West Region of Ghana, respectively “Fig 1”. Vegetation-wise, the area surrounding Agogo is largely covered by wet savannah grassland and partly by tropical rain forest [34]. The landscape surrounding Wa exclusively consists of open woodlands, grasslands and dry savannah [34,35]. Highly venomous snakes of medical importance in the areas studied include the saw-scaled viper (*Echis ocellatus*), the puff adder (*Bitis arietans*), the black-necked spitting cobra (*Naja nigricollis*) as well as the green and the black mamba (*Dendroaspis viridis* and *polylepsis*, respectively) [11]. While the saw-scaled viper predominates in the open woodlands and dry savannah of the North, the habitat of the green and the black mamba is confined to densely forested areas such as those surrounding Agogo [11]. The two geographically distinct sites covered in this study address regions with potentially different incidence rates of snakebite [5,10], snake species and variations in religious backgrounds and socio-economic differences.

### Study population and sampling

THs that practiced largely independent from one another were identified and approached through purposive sampling by local collaborators until data saturation became apparent. The initial predefined target was 30 THs (15 per location). During recruitment, special consideration was given to achieving a relatively homogenous geographical distribution of THs’ practicing sites, including some that practiced within the mentioned towns, but also some that practiced in rural villages considerable distances away (~ 42km). These criteria were applied to increase the possibility for heterogeneity of responses. Inclusion was based on the active practice of traditional medicine, including the supervision of snakebite patients during the year prior to the interview.

### Data collection

A semi-structured interview guide “S1 Appendix” was designed for THs covering the following topics: (1) personal and professional experience with snakebites, (2) knowledge of locally endemic venomous snakes, (3) symptoms of snakebite, (4) religious and spiritual interpretation of snakebite, (5) treatment of snakebite, (6) referral of snakebite patients to- and collaboration with hospitals, (7) social and economic consequences of snakebite, and (8) community education and prevention. If the TH was also a fetish priest, a slightly adjusted version of the interview guide which emphasized spiritual and religious aspects was used. The interview guide was tested during a pilot interview with a TH and finalized afterwards.

As far as possible, interviews were conducted at the practicing sites of traditional healers or other neutral venues to ensure an open and permissive discussion environment. The first author (JS) conducted all interviews apart from one, which was done by LA. Questions were asked in English and translated to the TH by an interpreter familiar with the local language and culture of the area. Answers by the TH were subsequently translated back into English by the interpreter and recorded for subsequent verbatim transcription by JS. Transcripts and recordings were marked using anonymized record numbers and stored on a secure server. Field notes were made to supplement audio data for individual interviews.

In addition to the interview responses, the following variables were collected: (1) sex, (2) age, (3) district and village, (4) religion, (5) years of practice, (6) other occupations, (7) education, (8) distance from nearest hospital (defined as either a nearby district/regional hospital or a hospital rendering in-patient care, but not a community health centre) in Kilometres (measured using google maps [36]), and (9) average number of snakebite patients treated in the rainy and dry season of the year preceding the interview. The listed variables were gathered and stored using REDCap (Research Electronic Data Capture). Demographic statistics were analysed using IBM SPSS Statistics version 26.

### Analysis of interview data

Interview transcripts were analysed largely in accordance with the methodology of thematic analysis as described by Braun and Clarke [37]. This decision was based on our objective to arrive at a broad and rich thematic description of a public health problem through the lens of local THs without testing a priori hypotheses or theories. The analysis of interview transcripts was carried out by two researchers. All interview transcripts were read once and a list of 62 codes was assembled. All data was coded, after which codes were collated into themes independently by the two researchers using ATLAS.ti 8 software. The resultant themes were discussed and unified. Any discordances were resolved in discussion with the principle supervisor. The ‘COnsolidated criteria for REporting Qualitative research’ (COREQ) were taken into account while writing the final report [38].

## Results

A total of 19 interviews were conducted, of which 11 were held in the area surrounding Agogo and 8 in the area of Wa. Interviews lasted an average of 84 minutes. Ten interviews were conducted at the home and practicing site of the interviewed TH, three at a local community health centre, four in public spaces/gathering spots and two at a local hospital. All but one of the THs interviewed were males. The median age was 59 (27–85) years without any notable differences per study location. Beside the practice of traditional medicine, the following occupations were also mentioned: fetish priest (5/19), farming (18/19), cocoa merchant (1/19) and singer/artist (1/19). All other demographic variables are shown in “Table 1”.

**Table 1:** Demographic characteristics and descriptive variables of THs per study location.

	Agogo n = 11	Wa n = 8
Education level No formal education Primary education Secondary education	3 4 4	4 2 2
Religion African Traditional Religion Christian Muslim	6 1 4	2 5 1
Median (IQR) years of practice	25 (8–28)	29 (18–44)
Median (IQR) number of snakebite patients treated during previous year Rainy season Dry season	5 (2–15) 3 (1–5)	11 (4–23) 13 (8–20)
Median (IQR) distance to closest hospital (km)	4.2 (0.5–38)	16.5 (4.1–34)

**Fig 2:**
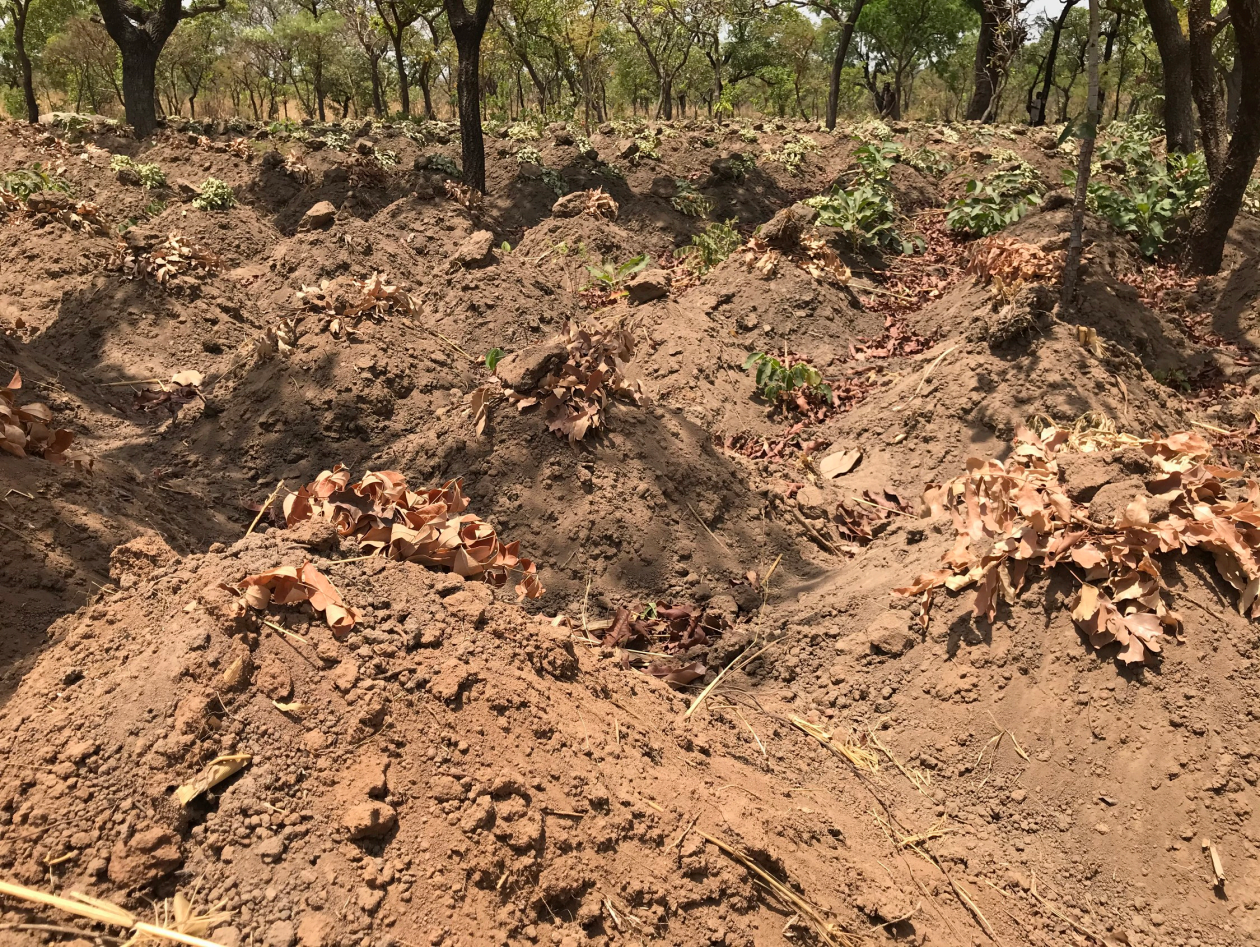
Yam mounds topped-off with leaves and branches to prevent desiccation of seedlings- a frequently reported hiding place of venomous snakes.

**Fig 3:**
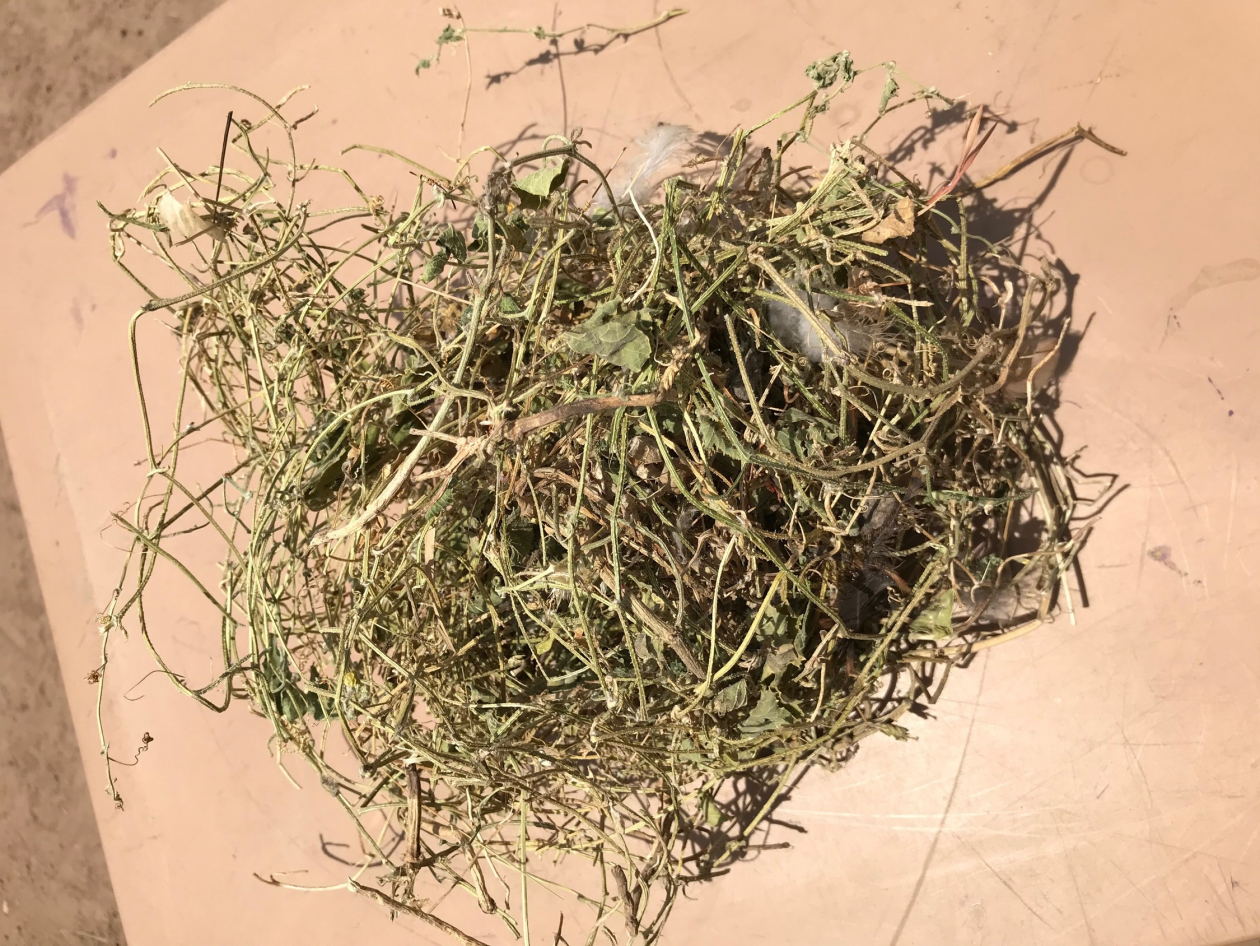
Herbs used in the treatment of snakebite.

**Fig 4:**
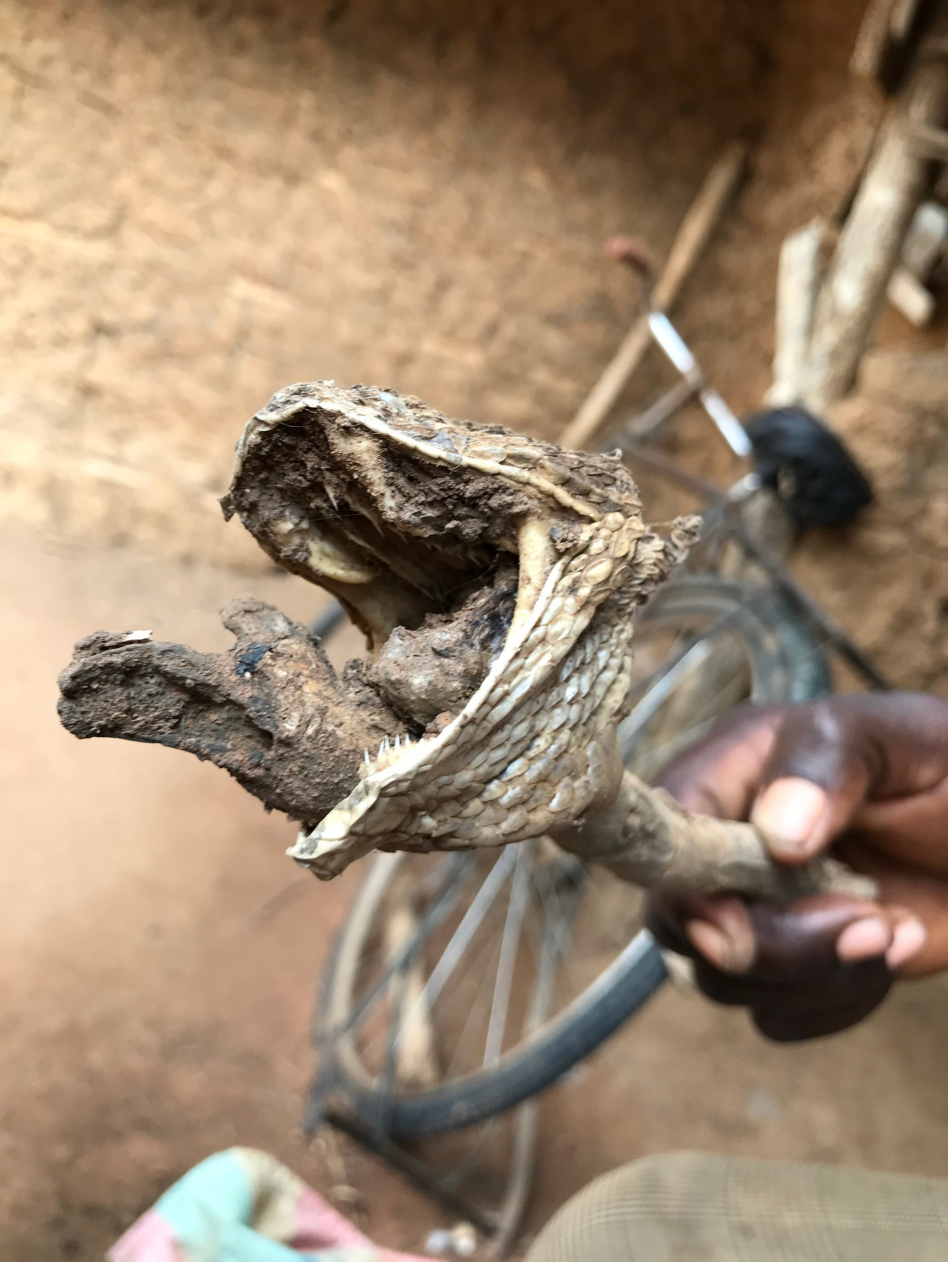
Head of a dead puff adder used as treatment ingredient by a traditional healer.

**Fig 5:**
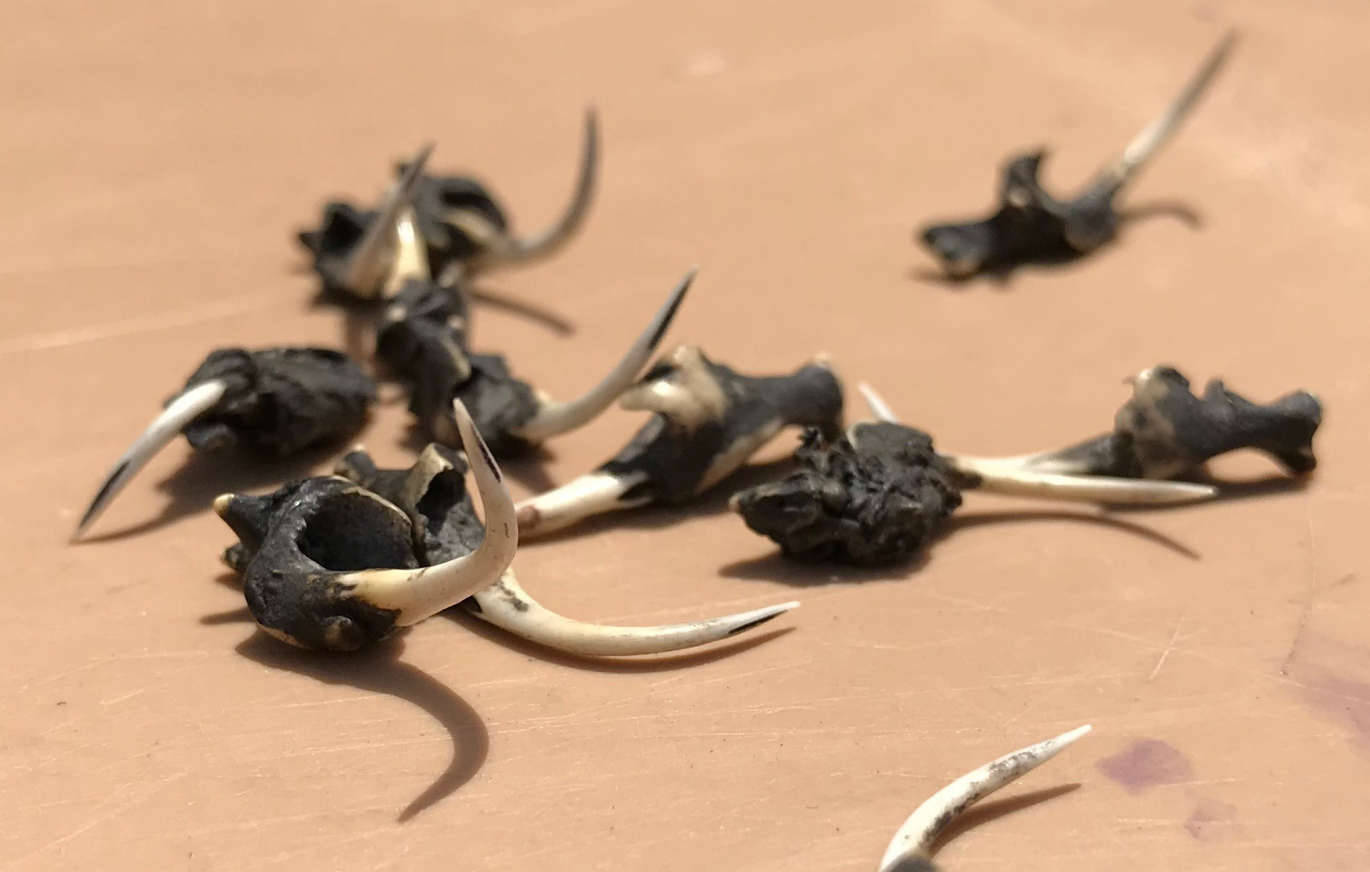
Snake fangs used to make incisions as part of traditional medical treatment.

**Fig 6:**
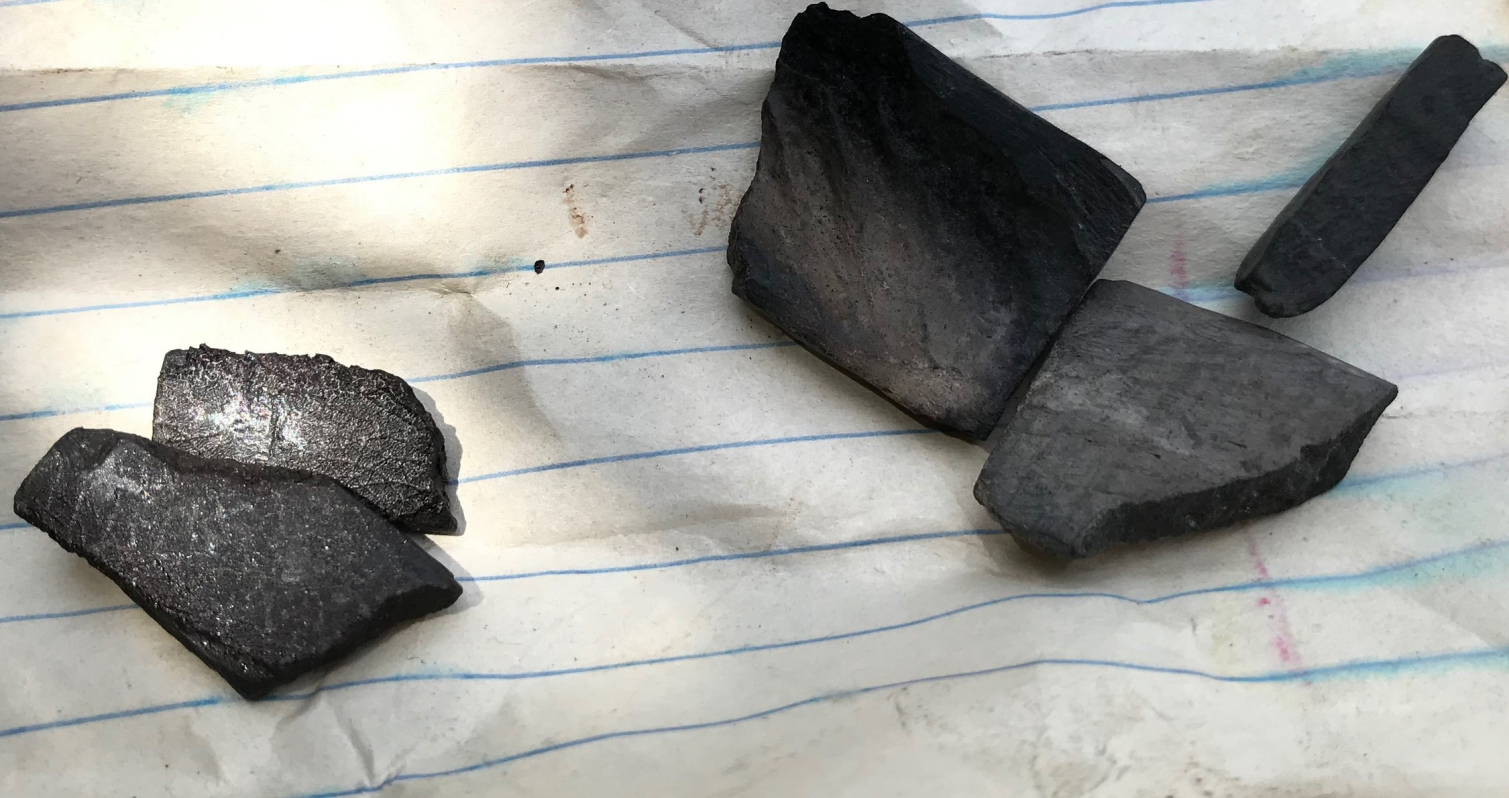
Black stones.

Images by J. Steinhorst, Upper West Region, 18.03.2020 (Figs 2–5) and Ashanti Region (Fig 6), 10.03.2020, Ghana.

A total of seven themes were derived from the interviews, which are outlined below, together with interview quotes that underscore the descriptions provided. Fetish priests were all THs as well, the only difference being their collaboration and consultation with spirits and deities. Hence, they are not discussed separately.

### Perception and knowledge of snakebite in the community: ‘They bite with reason.’

Snakes were ubiquitous in the rural communities where THs practiced, and exposure to snakes and snakebite was seen as inevitable by most community members that we engaged with. Over 60% of the THs interviewed (12/19) had experienced a snakebite personally, mostly on the farm “Fig 2”. Commonly named secular risk factors were farming, hunting, collection of firewood and walking in the dark. Women who engage in shea nut picking and children curiously exploring their environment were particularly singled out as risk groups. Moral wrong-doing, such as stealing or physical abuse were linked to snakebites as well.

“*You anger it [Bitis arietans] mostly by stepping on its tail. You could step on any other part of the body and it could be cool, but on the tail, you anger it.” TH9, Male*

“*It can be related to a curse*, *if you are cursed*, *you can have a bite*.*” TH16*, *Male*

“*They bite with reason*.*” TH3*, *Female*

THs were nearly unanimous in their belief that all snakes are venomous, albeit to varying degrees. When shown pictures of five highly venomous snakes native to Ghana, some snakes were persistently seen as dangerous, whereas others were brushed-off as harmless.

“*He* [*Naja nigricollis*] *spit*, *you can go blind and you can also die when it bites you*.*” TH1*, *Male*

“*No*, *it* [*Bitis arietans*] *won’t bite*, *it’s the most foolish of all snakes*.*” TH4*, *Male*

“*No*, *it’s* [*Dendroaspis viridis*] *not very dangerous*.*” TH16*, *Male*

### Acquisition of knowledge of traditional medicine and treatment practices: ‘The spirit just came upon me.’

THs’ knowledge of traditional medicine and treatment practices in snakebite was almost exclusively acquired from predecessors (grand-fathers, fathers) or from mentors (THs) external to the family through ‘apprenticeships’. Once a family member, mostly within the nuclear family, has the knowledge and skills to treat the diseased, the children are acquainted with the herbs and ingredients used to treat certain ailments. The children’s role is not insignificant, often being sent to gather the required herbs in the surrounding bushland.

“*It’s* [the knowledge] *handed over from my great-great grandfather to my grandfather*, *through my father and to me*, *so I learned it from this lineage*.*” TH17*, *Male*

“*My grandfather introduced me to the herbs*, *so anytime somebody came to the house with a snakebite*, *I was asked to go to the forest and bring the herbs and then my father will mix it with something* […] *to manage the patients*.*” TH1*, *Male*

External mentorship and training within the family were either the sole source of knowledge or they were coupled to a divine calling. It is believed that traditional priests are gifted with the divine ability to follow the guidance of deities and spirits to treat victims who are afflicted with various ailments, including snakebite.

“*The spirit just came upon me*.*” TH3*, *Female*

“*The gods have called me to help society*.*” TH7*, *Male*

Some claimed that there were occasional gatherings at which hospital workers and THs discussed health-related issues or where THs received treatment instructions. Some THs, particularly those stationed within or close to towns, reported to attend professional gatherings of THs for updates. Advice-seeking from senior mentors (THs) was also a source of updates. On the contrary, the practice of traditional medicine appeared to be subject to competitiveness as well, highlighted by a wide-spread reluctance to divulge the ingredients of concoctions and the claim that competitors may even be out to deceive others.

“*There is a challenge amongst us* [the THs], *everybody wants to prove they are powerful*.*” TH4*, *Male*

The request for more teaching and learning offered by the hospital was expressed as an opportunity for improving practices and deepening ties with the healthcare sector.

“*I would like to learn from the hospital and add to my practices to improve on my practices and also get more knowledge about the treatment*.*” TH5*, *Male*

“*It would be helpful if we had a good collaboration with the hospital*.*” TH7*, *Male*

### Health seeking behavior of snakebite patients: ‘They’ve taken me as a spiritual father.’

THs generally considered themselves to be well-known and trusted within their communities and beyond, also by healthcare workers in some cases. As illustrated below, THs can be central figures within the social and political constructs of communities, providing social leadership and counselling.

*“They* [the patients] *appreciate me so much and they*, *more or less they’ve taken me as a spiritual father and if someone has any complicated issue*, *aside snakebite*, *quickly*, *they come to me*.*” TH8*, *Male*

Patients were said to either present immediately after the bite or up to days, weeks or at most a month later. A recurring issue within the theme of health seeking was in fact the delayed presentation of patients at THs’ practices. Issues noted with regard to delayed presentation were the insidious nature of some snakebites, going unrecognised at first and later manifesting through symptoms such as swelling of the bite site, or the great distances victims had to cover in order to seek help. One TH noted that he travels outside of his community to ensure timely treatment of snakebite victims. The delay in health seeking was recognized as a problem and associated with worse clinical outcomes and death.

“*It doesn’t really take days*, *because if it takes days*, *it will bring about mortality*, *yes*.*” TH3*, *Female*

“*So the biggest challenge is that some don’t report early*. *Some don’t report so early*, *they stay in the villages for long*, *they come with very big ulcers*.*” TH3*, *Female*

Snakebite was widely perceived as an acute emergency with potentially deadly outcomes.

“*It doesn’t bite for you to leave; it bites to kill*.*” TH8*, *Male*

Many snakebite consultations of THs were however not necessarily borne out of emergency situations. Traditional medicine was said to also be possibly sought in conjunction with or after treatment at the hospital. The primary concerns of patients were supposedly unsatisfactory treatment at the hospital, the high costs associated with the latter and neglect of spiritual aspects related to the bite.

“*Here it’s not like a doctor where you go*, *if you don’t have money*, *you will not be treated*.*” TH1*, *Male*

“*Some do go to the hospital and come back here*, *because the intensity of pain is too much*. *And others too come*, *because the swelling is getting intense*.*” TH2*, *Male*

“*Some will abandon the hospital treatment and come to me for treatment*, *which of course is certainly cheaper as compared to buying anti-snake venom*.*” TH2*, *Male*

The handling of money in relation to treatment and traditional medicine was suffused with symbolism and even taboos for certain healers. Money was reported to spoil the effects of traditional medicine and many preferred gifts or to be paid in kind (e.g. fowls, goats, drinks, corn, bread and cloth) or even the manual labour of the patients over money.

“*The medicine is not for sale*, *if I do collect money*, *then I’ll spoil it*.*” TH13*, *Male*

“*It can be sheep*, *it can be goat*, *can even be a cow*, *depending on your strength*, *you the patient strength*. *Yeah*, *then plus 700 cowries*.*” TH12*, *Male*

Nearly a third (6/19) of the interviewed THs indicated that the high costs associated with hospital treatment reduced its accessibility for patients and some healers even reported that patients struggled to compensate for the comparably affordable traditional therapies.

### Recognizing snakebites: Pitting oedema and ‘sugar cane’ skin

The diagnosis of snakebite in the traditional medical setting, besides characteristic clinical symptoms, was said to be based on anamnesis, physical examination and consultation with dwarfs and deities. The reported differential diagnosis of snakebite only included scorpion bites. Causality was reported to be obvious in cases when victims brought the dead snake to consultations, but was more complicated in other cases.

“*[I] press to see whether there is depression*. *Yes*, *try to see if the person is oedematous*.*” TH16*, *Male*

“*If I apply that herb and if it’s bitten by a cobra*, *the site becomes dark*, *or if it’s any other type*, *the place swells up*, *or the contours become like that of a sugar cane*.*” TH4*, *Male*

“*But if you really doubt the snake that bite you and the charmer is there*, *he can call the snake*.*” TH12*, *Male*

“*I can detect the type of snake from the smell and the smell differs from snake to snake*. *The scent of the various snakes are like some trees*, *for instance*, *and then some ants*, *some big ants*.*” TH12*, *Male*

In cases where a “spiritual bite” is suspected, which was most often believed to be related to the patient not responding to ordinary treatment, divine consultation was deemed necessary. Four THs who also served as fetish priests explained they were capable of such consultations, while three THs said they referred patients to fetish priests for such matters.

“*I don’t do the consultation*, *but they’ll go and consult gods- I mean the fetish- to know what the person has done wrong*.*” TH12*, *Male*

One healer even raised the possibility of determining a spiritual cause of the bite using the dead snake.

“*Yeah*, *I can easily distinguish*. *And this is done by*, *if it is a spiritual I can easily see all the signs and symptoms*, *if it is a normal snakebite I can also see it*, *so the spiritual one*, *when it happens*, *I kill the snake*, *open the abdomen*, *and you will see that there is no intestines*. *If there is no intestine it means it was sent by a spiritual force*.*” TH1*, *Male*

If the patient did not respond to treatment, this also raised the suspicion of a spiritual bite. In case the snake was neither brought, nor seen, charming of the snake, consultation with the ‘oracle’ or sniffing of the bite site were employed for diagnostic purposes.

### The traditional treatment approaches: ‘I remove the teeth.’

The traditional treatment repertoire discussed consisted of herbal concoctions, physical interventions, prayers and sacrifices. Concoctions were said to contain combinations of any of the following natural products: herbs “Fig 3”, leaves, tree roots, bark, lemon juice, eggs, shea butter, dwarf tobacco and animal parts from snakes “Fig 4” and birds. These ingredients were mixed, burnt, mashed or ground to form a powder or homogeneous liquids.

“*Yes*, *so what I use ‘Bosre’* [viper] *for*, *the head of the ‘Bosre’*, *I use it to even prepare the medicine*.*” TH13*, *Male*

Such concoctions are applied locally to the bite site or are ingested systemically either alone, with a meal (e.g. porridge) or with a beverage (e.g. tea, schnapps/strong alcohol). The efficacy of ingested concoctions was explained by some through ‘neutralization of the venom’ or detoxification through emesis.

Interventions that were frequently reported to be performed at bite sites included removal of the fangs (or invisible fragments thereof), incisions “Fig 5”, less commonly the application of the black stone “Fig 6”, tourniquets and wound care using latex gloves.

“*I remove the teeth*, *I use a substance*, *like chewing gum*, *always starchy*, *then I will send it closer to fire and put it on where the snake bit the client and remove the teeth and most often*, *four teeth are only left in the system of the human being*.*” TH14*, *Male*

“*This one* [*Naja nigricollis*] *is very venomous*, *very poisonous*, *so after the bite*, *you should apply a tourniquet*. *If you don’t*, *the venom will climb up and could cause some life-threatening emergencies*.*” TH3*, *Female*

“*What I do most is that when they come with wounds*, *I go to buy gloves*, *so that I wear on my hands and take good care of the wound for them*.*” TH3*, *Female*

Immediate washing of the eyes after attacks by black-necked spiting cobras was moreover recommended using plant extracts or urine.

“*Right away*, *you have to urinate into your palm and then wash your eyes with it*, *fast*, *by the way*.*” TH10*, *Male*

While wearing boots, gloves, other protective clothing and certain behaviours were identified as preventative measures against snakebite, a widely practiced method to prevent snakebite was the ingestion of herbal concoctions as a means of fortification against snakebites. Such concoctions are thought to prevent snakebites, render them less dangerous and to even cause death of any surrounding snakes.

“*… I mean if you drink that medicine it fortifies you for at least six months*, *so within the six months you cannot have snakebite*. *After six months you will need to repeat the dose*, *so it’s like immunization*.*” TH13*, *Male*

### Contraindications, referrals, and complications: ‘If it’s spiritual, it’s my limit.’

For certain procedures THs also cited contraindications that guided them in their practice. This suggests that a few THs have acquired some physiological and anatomical concepts similar to biomedical theories useful in avoiding the exacerbation of patient’s symptoms. Many healers, however, did not express such explanations.

“*I use only the herbs*, *I usually don’t like using other interventions like making marks with razor blades*, *because probably the person might have- might be diabetic and those cuts may cause problems*, *or you don’t even know the problem that the person- the other sickness the person has*, *the other conditions- medical history of the person*.*” TH5*, *Male*

Despite the general tenor of confidence in their own treatment methods, there were also some challenges acknowledged by THs.

“[The] *greatest challenge is when the whole leg is swollen and it’s dripping with fluid and all that*, *that’s the greatest challenge*. *Or when you see total weakness*, *the patient is like paralyzed and all that as a result of the bite*, *that is a great challenge too*.*” TH8*, *Male*

“*Yes*, *if it’s spiritual*, *it’s my limit*. *If the person also* [becomes] *anaemic*, *it’s my limit*, *I have to refer to the hospital*.*” TH13*, *Male*

Referrals were moreover considered for tetanus shots, antivenom injections, management of cellulitis and infective complications or cases where mortality appears to be imminent to the healer.

“*After seeing him* [the patient], *I refer apart from the tetanus*, *so that they can be checked for also other infections that may come up or that stuff*.*” TH5*, *Male*

On the long term, snakebites were associated with ulceration of the bite site, persistent swelling and pain, wound infections, ‘shivering in the rain’, loss of teeth, rotten tissue, deafness, weakness, deformities and amputations.

“*It can lead to amputation*, *if you don’t take care*, *they can amputate the whole leg*.*” TH13*, *Male*

“*But even after the treatment*, *the leg was not straight*. *The mother’s leg*, *where the snake bit*, *was not straight again*.*” TH12*, *Male*

Four THs acknowledged that snakebite patients had died while being on their treatment. Two healers explained that the deaths had spiritual connotations and the other two claimed that patients had not adhered to the prescribed treatment.

### A common goal: ‘The main objective is to get the patient cured.’

Knowledge of procedures at the hospital ranged from tetanus injections, antivenom administration, blood transfusions and infusions to amputations. Nearly all (17/19) interviewees rated antivenom as being effective.

“*Yeah*, *it* [the antivenom] *really helps and I believe it’s a very good remedy and if we are to rank it*, *it’s the best*.*” TH8*, *Male*

Criticism of hospital therapies were primarily related to the belief that antivenom is only effective as an immediate therapy (3/19) but not on the longer term. Moreover, around half (9/19) of THs repeatedly emphasized that healthcare workers at the hospital did not remove snake fangs from the wounds.

“*Antivenom is effective, but in the hospital they cannot remove the fangs, or they do not remove the fangs so they only give you the antivenom which will solve the problem immediately, but in later years to come, you feel the symptoms can resurface. For instance chills and then other things, symptoms.” TH16, Male*

Collaboration and communication with health facilities was seen as important by most interviewees and many said they would welcome better relationships and more exchange of information. This applied to feedback on referred patients and to antivenom, the presence of which was mostly known through patients who attended clinics and returned.

“*Some* [patients] *go and they come back to tell*, *‘When I went*, *I had anti-snake venom’*. *Some also go and come and they tell ‘No*, *I didn’t have any’*.*” TH3*, *Female*

The opinion that collaboration and improvement of ties between health facilities and THs is necessary was not shared by all, though. An explanation offered towards this stance was the distinction between ‘hospital-based’ and spiritual aetiologies of disease.

“*Some of them are hospital-based. But the other diseases, that doesn’t need to be at the hospital. More especially diseases that have spiritual implications attached can be managed here, not at the facility level.” TH1, Male*

“*We are parallel, they are working, I am also working. I don’t have anything against them, they don’t also have anything against me, but there is no way there is any connection where we need to really do anything too.” TH12, Male*

## Discussion

Through interviewing THs in snakebite endemic communities, we tapped indigenous knowledge and beliefs on the subject and brought to light the concerns of healers and community members on the matter. Prior studies on health seeking and traditional medicine in snakebite have examined the subject mostly from the community or the hospital perspective [18,20,39] focusing on those seeking traditional care, but rarely including accounts from those providing traditional care [10]. Our study echoes calls for more ‘ethno-anthropological’ [4,40] research in snakebite-endemic regions and conforms with the doctrine of a ‘biosocial’ approach to healthcare, as brought forward by Farmer et al. [41].

It readily emerged from the interviews that communities where THs practiced experienced a high burden of snakebite. All THs indicated they had treated several snakebite victims in the year prior to the interview. In addition, numerous anecdotal reports about snakebite incidents, the detailed descriptions of treatment practices and the high proportion of THs (12/19) who had personally experienced a bite all suggest a high incidence. Community surveys are urgently required to corroborate these findings and to elucidate the reasons that a fraction of patients do not present to hospitals [8]. The risk factors and activities associated with snakebite, such as farming, shea-nut picking and hunting mostly aligned with those reported by others in the region [10,24,26]. A striking finding was the often described relatively dichotomous categorization of the bite cause as being either ‘spiritual’ (i.e. related to moral wrong-doing) or ‘physical’ (i.e. accidental) in nature. While many THs mentioned that distinguishing the cause was an important prerequisite in choosing the correct therapy and making a prognosis, the implications hereof might extend beyond the medical field. The widely held belief that bites can be incurred for spiritual reasons supposes that the threat of snakebite plays a role in maintaining social order and motivating good moral conductance of community members [27]. Being so charged with symbolism and spiritualism invariably makes snakebite a challenging health issue to address, for which holistically geared medical and public health measures are more likely to succeed than purely biomedical ones [40].

From their own point of view, THs appeared to enjoy an uncontested status as primary healthcare providers within their communities, a valued prominence for most THs. Community surveys and hospital data from a neighbouring district and other countries showed that traditional medical practitioners were indeed sought in 40–63% of cases [10,18,39]. Affordability has often been brought forward as a pivotal factor leading to the high acceptance rates of traditional medicine [14,27,28]. The healers interviewed for this study reported themselves to not ask much of their clients for compensation. Up-front payments were the exception and reimbursement in kind in proportion to the patient’s ability or the expression of mere gratitude were mostly judged as sufficient. Money was even outright rejected by some healers because including financial compensation whilst engaging in an exchange involving medicine conflicted with their beliefs and was said to ‘spoil the medicine’ [42]. This was in line with the professional motivation stemming from a divine calling, as expressed by some.

It could be deduced from the interviews that the process of health seeking was neither bimodal (either hospital or TH), nor unidirectional (e.g. first TH, then hospital). Although THs were the first point of call in the village, this did not preclude subsequent visits to hospitals or the parallel use of allopathic medicine and traditional therapy, even when distances to hospitals were considerable. A major issue identified by THs was the delayed health seeking of patients after having incurred a snakebite. This observation underscores that bottle necks to seeking help, such as long distances and lack of transportation, are also frequently encountered in patient’s pursuit of traditional medical therapies. The relevant literature describes such obstacles as being more commonly associated with delays in seeking hospital care, whereas traditional medicine is thought to be easily accessible in comparison [14,18,25,39]. Antivenom was widely considered as effective by healers and was a primary reason for referring patients to hospitals. This was noted despite that fact that many antivenoms used in Sub-Saharan Africa are unspecific to regionally endemic snakes and/or have questionable clinical efficacy and safety profiles [17,43]. Patient movement from hospitals to healers, on the other hand, was mostly explained by the suspicion of a ‘spiritual’ bite, persisting pain, unavailability of antivenom and insufficient treatment. Taken together, this pattern of consultation is probably borne out of patient’s needs to address psychological suffering linked to strong beliefs about spiritual causes of the bite, alongside the alleviation of clinical syndromes like pain, swelling and paralysis [27]. This is also in line with the finding that snakebite patients in Sri Lanka who had relatively good access to allopathic healthcare still opted for traditional therapies [18].

Knowledge acquisition on snakebite therapies was almost exclusively from family members, as is common in the practice of traditional medicine [21,24,30,42], or, rarely, other members of the community and meetings with other THs. The lack of formal and standardized training was reflected by the vast array of different diagnostic as well as treatment methods reported by THs [28]. Limited training, restricted sharing of knowledge and poor collaboration among THs, but also between THs and medical staff most certainly has a negative impact on morbidity and survival of bite victims [11,20,39]. This is compounded by insufficient transparency by many healers about their methods [27], but also their outcomes, which complicates any effort at scientific scrutiny and assigning responsibility for adverse treatment outcomes. The open stance taken by many healers towards hospital medicine, however, signifies that there is potential for finding common ground between THs and clinical health staff in addressing the burden of snakebite. Closer association between THs and medical facilities and training of healers is widely considered as vital to the improvement of healthcare [11,13,14,40] and research has already demonstrated that such progress is possible and worthwhile for other ailments [44–46]).

While biomedical treatment of snakebite is based on a syndromic approach [11], many THs ascertained that they were able to differentiate offending snakes and even the cause of the bite using a variety of methods. We found that several THs had difficulties distinguishing non-venomous and venomous snakes, often labelling highly venomous snakes as harmless. Likewise, the alleged diagnostic power of topical herb applications to the bite site with subsequent blackening of the skin as an indicator of snakebite may be based on necrosis, which is a consequence of cytotoxic venom components. These obvious knowledge gaps of THs underline their dire need for training and education on the correct identification and diagnosis of snakebite.

Similar to what other authors have reported [20,24,39], treatment included ingestion or topical application of plant and animal-based concoctions and manipulations of the bite site. Manipulation of wounds for purported fang removal and incisions for blood-letting and snake-stone application can exacerbate bleeding and cause wound infections [20], in the worst case with *Clostridium tetani* [47]. Tourniquet application, reportedly used in cobra bites by a TH in our sample, can exacerbate tissue necrosis and swelling, thereby increasing chances of later amputation [20]. The most common adverse effect of traditional therapies, however, is probably related to the delay it introduces until patients finally seek hospital care [13,20,39]. Nevertheless, concoctions were frequently said to be mixed with liquor or spirits which may have a positive effect on the level of pain experienced [48], albeit not necessarily being an effective treatment to prevent or reverse the venomous effects of snakebites. The use of lemon juice makes for an interesting case. Lemon juice has been described as having protective effects in snakebite for thousands of years [49] and *in vitro* and *in vivo* anti-haemorrhagic activity has been demonstrated in a study on *Bothrops atrox* toxicity in Columbia [50]. Moreover, lemon juice (i.e. citric acid) is part of commercialised wound care products that are used clinically [51,52].

The explanations for some therapies were grounded in physiological and anatomical concepts similar to biomedical theories. Treatment was for example meant to ‘neutralize’, ‘drain’ or prevent spread of the venom, proving that many THs agreed with the biochemical concept of envenoming [20]. A further example is the advice to flush the eyes with urine after spitting cobra attacks. Likewise, one TH cautioned to not make incisions on people for fear of complications arising from existing co-morbidities, such as diabetes and another claimed to treat patient’s wounds using latex gloves. These examples illustrate the scope and diversity of traditional medical approaches in snakebite and that some THs are in parallel with certain aspects of hospital practices.

The high success rates and the alleged effectiveness of traditional treatment measures seem questionable at first sight if compared to clinical syndromes of envenoming and the morbidity and lethality of snakebites as reported in literature [7,9,10,25,53]. For example, the lethality of bites by *Echis ocellatus*, a deadly and very common snake in West Africa, is estimated to lie between 12–20% in the absence of antivenom therapy [7,26,53]. But there are some arguments in support of THs’ optimistic stance. One is the high proportion of both bites by non-venomous snakes and dry bites, i.e. bites that do not involve venom injection by an otherwise venomous snake [8,54,55]. As no envenoming took place, recuperation after a dry bite would be speedy and would falsely be ascribed to the therapy given [20]. The other argument is forthcoming from the interviews; several healers reported to refer patients that had experienced severe grades of envenoming to avoid recording any mortality. In support of this argument, a study in Sri Lanka found that clinical syndromes of envenoming (i.e. obvious envenoming and no dry bite) were indeed associated with health seeking at hospitals [18]. In this case, what may benefit the reputation of the healer may strain that of the hospital, creating the misconception among the public that hospitals are unable to treat snakebite patients. In a similar vein, the four patient deaths admitted by THs in this study were either ascribed to spiritual factors or to treatment default by the patients, but not to ineffective therapy. In fact, if traditional therapy was ineffective it was widely believed that there was a spiritual cause behind the bite. Such explanations are not unique to snakebite and are also not new in the context of health and disease in West Africa [27]. The interplay between all these factors may ultimately culminate in a positive feedback loop reinforcing health seeking at THs [13].

### Strengths and limitations

The research framework applied enabled the collection of qualitatively good data. Healers were interviewed in two geographically and ethnically distinct regions and considerable geographic spread of healers was achieved within regions, providing a large breadth of responses. The sampling methods aimed at including healers of different religious backgrounds, as well as those also active as fetish priests. Two thirds of all interviews were held at local homes or villages of THs, ensuring a high likelihood of unbiased responses.

Nonetheless, we also acknowledge shortcomings. First of all, the targeted sample size of 30 THs could not be reached. Data collection was prematurely aborted at the end of March due to travel restrictions as measures against the SARS CoV-2 pandemic took effect. The decision to still proceed with the analysis was based on data saturation becoming noticeable. Secondly, interview quality was occasionally compromised through bystander commentary, interpreter bias and potentially also by the venue (e.g. hospital or health centre). In one instance the interpreter remarked that the interviewee was reluctant to reveal information for fear of reprisals or correctional measures following the interview.

## Conclusion

We found that the most important qualities of THs in relation to health system strengthening were (i) that THs seek greater engagement with hospital/health facilities to improve the outcomes for their patients, (ii) that their primary goal was therapeutic and not financial and (iii) their identification of long term snakebite sequelae that arises from prolonged contact with victims that is very rare with conventional healthcare professionals. However, this research also brought to light the following drawbacks of traditional medicine: (i) the misidentification of highly venomous snakes as being harmless, (ii) the lack of standardization of treatment methods, education and training, (iii) the potentially harmful treatment practices associated with delay in seeking clinical care (iv) and (v) the insufficient transparency about treatment practices and denial of responsibility for adverse outcomes.

There are currently few channels of communication between THs and healthcare facilities to the disadvantage of patients, who for example go searching for antivenom in vain. This underscores the need for updating THs on the availability of antivenom while concomitantly improving its stocking, quality and affordability. Health seeking would likely improve as a result since antivenom was widely regarded as effective by healers. Besides improving the availability of antivenoms, clinical care requires effective pain management and wound care, which has a high potential of increasing clinical treatment adherence and health seeking at the hospital level. At the community level, training and education in first-aid practices should be offered to THs who are willing to participate. Healers and the general public are also likely to benefit from education campaigns explaining the behaviour of snakes, clinical syndromes of envenoming and the best possible conduct and first-aid measures following a bite. A combination of these efforts is urgently needed to increase health seeking of patients at hospitals and to reduce snakebite related morbidity and mortality.

## Supporting information

S1 Appendix Interview guide for traditional healer. (DOCX)
