# Supplementary material for: ‘The medicine is not for sale’: Practices of traditional healers in snakebite envenoming in Ghana
Source: PLoS Negl Trop Dis. 2021 Apr 16;15(4):e0009298. doi: 10.1371/journal.pntd.0009298 (PMC8081335; doi:10.1371/journal.pntd.0009298)
Supplement: S1 Appendix — (DOCX) [file pntd.0009298.s001.docx]

**S1 Appendix: Interview guide for traditional healer**

Note: These questions were used to stimulate an in-depth conversation with the TH on a range of topics and were not asked in the style of a simple questionnaire. Questions were grouped under five main sections to cover the eight topics listed in the ‘Methods’ chapter of the manuscript: 1) Experience, 2) Treatment, 3) Referral and collaboration, 4) Social and economic consequences of snakebite and 5) Community education. The sequence in which these five topics were discussed was varied during different interviews.

**Experience**

What do you know about snakebites?

What is your experience with snakebites in your own living community?

What is your experience with snakebites in your work as a healer?

How did you acquire your knowledge on snakebite treatment and management?

Have you ever been bitten by a snake yourself?

How do you recognize a complaint as a snakebite?

- 1. What are the signs and symptoms you look out for?

What do you do in case you are not certain it was a snakebite?

At what time point do snakebite victims visit you after they had the bite (minutes, hours, days)?

Do snakebite victims treat themselves prior to seeking the help of the traditional healer?

1. If yes, what kind of self-treatment?

Do patients also consult you after they visited a clinic/ hospital?

1. If yes, why do patients come back to you?

What do you tell snakebite victims when they visit you?

1. Do you explain how the bite happened, why the bite happened?

Can one possibly distinguish between purely bodily symptoms and spiritual symptoms?

Is there any influence or relationship between snakes and witchcraft?

1. If yes, could a witch also treat snakebite victims?
2. Could a snakebite be related to a curse?

Are snake charmers used to find the snake that bit the victim?

1. If yes, is this the same person as the traditional healer or is it someone else?
2. How would they catch the snake? And why?

Are all bites poisonous?

1. If no, can you tell whether venom was injected or not?

Show pictures of 5 types of snakes*,* saw-scaled viper, puff adder, green mamba, black mamba and black-necked spitting cobra. Questions for all the pictures:

1. What do you know about this snake?
2. Do you think this snake is dangerous?
3. How would you treat a patient bitten by this snake?
4. How do you call this snake in your local language?
5. Which of these snakes cause most bites in this area?

Besides these snakes, are there any other kind of snakes that commonly bite people in this area?

1. Can you name any such snakes?

**Treatment**

What kind of treatment would you give to snakebite patients?

Does the treatment depend on the type of snake?

If yes, how do you identify or differentiate the type of snake that bit the victim?

And how do you choose which medication to administer?

Do you give snakebite victims herbal medicine?

1. If yes, what kind of herbs?

Do you recommend them to use a black stone?

1. How do you use the black stone?

What is the name of a black stone in twi/other languages?

1. Where do people get the black stone from?

Do you ever feel that you have reached a limit with your methods?

1. Are there methods others are using that are more/less successful?

Do you give your snakebite patients any other treatment or intervention?

How long does is take you to treat a snakebite patient?

1. What do you define as ‘treatment completed’?

Do you know other traditional healers who treat snakebites?

Are traditional healers who treat snakebite victims male or female or does it not matter?

Is every traditional healer able to treat snakebite victims? Or are there traditional healers that are specialized in treatment of snakebite victims?

Are there any differences in the treatment of snakebite victims between you and other traditional healers? Or is the same treatment used by all traditional healers?

Are there people other than the traditional healers that treat snakebite victims?

1. Are these people consulted by the snakebite victims?

Do you help catch snakes?

1. How?

What is the biggest challenge in the treatment of snakebite victims?

**Referral and collaboration**

How do you advise patients after you treated them?

1. Do you advise them to go to a health facility?
2. If yes, when and which health facility?
3. Are there any clinical signs that make you decide to refer a snakebite victim to a healthcare facility?
4. Do you follow-up on patients?
5. Have you ever lost [death of] a patient during treatment? What happened?

How is your relationship with the healthcare facilities (hospitals) in your area?

- 1. What perceptions do you think the people working at the hospital have about your treatment methods and approach to snakebite?
  2. What perceptions do you think the community members have about your treatment methods and approach to snakebite?

Could something be improved in the collaboration between you (the traditional healer) and the healthcare facilities?

1. How?
2. Do you get updated about the availability of antivenom at local hospitals and pharmacies? Where do you get that information from?
3. Is antivenom effective at treating snakebite?
4. How could referrals from you to the hospital be made easier?
5. Is motorized transportation available in your village/ neighbouring villages?
6. Have you heard of treatments at the hospital that you would like to be able to perform yourself?

**Social and economic consequences of snakebite**

On average, how much do you charge for treating snakebite victims?

1. Are there any differences per snake type or does it depend on the complaints victims come with?
2. If yes, why does it vary?

Do you accept part payment and/or treat on credit bases?

Do you accept any other forms of payment?

1. If yes, what kind?

What do you think are the long-term consequences for snakebite victims in daily life?

1. What could be improved for them?
2. Is there anything a traditional healer can do for the victims after the initial treatment to reduce complications and long term consequences?
3. If so, what?
4. Do you counsel victims of snakebite who suffer from psychological trauma after the incident?

How do snakebites affect patients socially and economically?

1. What should be improved?

**Community education**

Do you think snakebites can be prevented?

1. How?

Do you think education on the prevention of snakebites is needed?

1. If yes, which prevention strategies would you highlight?
2. If no, why not?

Do healthcare facilities provide enough information and education about snakebite to the community?

If you would educate people on the prevention of snakebites, what would you tell them?

How do you stay updated about changes in your profession e.g. regarding information about snakes/snake venom and the treatment?

Do traditional healers inform each other based on experiences in treatment?

Is there anything you would need to improve in the way you take care of snakebite victims?

1. If yes, what would you need?
